# Editorial: Cross-talk and interaction between endocrinology and urology: challenges and opportunities

**DOI:** 10.3389/fendo.2023.1269484

**Published:** 2023-08-21

**Authors:** Yuxuan Song, Caigan Du, Xiaoqiang Liu

**Affiliations:** ^1^ Department of Urology, Peking University People’s Hospital, Beijing, China; ^2^ Department of Urological Sciences, University of British Columbia, Vancouver, BC, Canada; ^3^ Department of Urology, Tianjin Medical University General Hospital, Tianjin, China

**Keywords:** endocrinology, urology, andrology, prostate, adrenal

The urinary and reproductive systems are closely interconnected with the endocrine System (1, 2). Some of the urinary and reproductive organs themselves function as endocrine organs. If these organs undergo pathological changes, it can affect the endocrine system (3). Conversely, when the endocrine system experiences metabolic disorders, the urinary system can also be affected (4, 5).

In recent decades, there have been rapid advances in the diagnosis and treatment of endocrine and urological diseases. Therefore, this Research Topic aims to study the endocrine metabolic disorders in patients with urinary and reproductive system diseases and their pathophysiological mechanisms. Through this interaction, we hope to promote cooperation and communication among experts in the fields of urinary and reproductive systems and endocrinology, and to carry out meaningful collaborative research, thereby proposing new ideas for diagnosis and treatment.

Liu et al. investigates the impact of steroid hormones on retinal neurodegenerative diseases (RND) using genetic variations as instrumental variables. Glaucoma risk was found to be influenced by testosterone/17β-estradiol (T/E2) (OR = 1.11, 95% CI, 1.01–1.22, P = 0.03), which was validated by multiple methods. However, no impact of other steroid hormones (aldosterone, androstenedione, progesterone, and 17-hydroxyprogesterone) was observed on diabetic retinopathy (DR) and age-related macular degeneration (AMD) risk. The study suggests that T/E2 may have a suggestive effect on glaucoma risk, but further research is needed to explore steroid hormones as targets for prevention and treatment.

An et al. investigates the relevance of metabolic syndrome (MetS) and metabolic scores to metastatic prostate cancer (PCa) occurrence, progression, and prognosis. Patients with MetS had higher T stage, Gleason score, and tumor load, with shorter time to progression to castration-resistant PCa stage. The median survival time was significantly shorter in the MetS group. Metabolic score correlated with survival time. The study concludes that MetS may promote metastatic PCa progression and affect prognosis, suggesting its role as a risk factor in metastatic PCa outcomes.

Li et al. evaluates the outcome and safety of retroperitoneal laparoscopic partial adrenalectomy in treating nonfunctional unilateral adrenal tumors in the day surgery mode. The surgery was performed on 19 patients, and the results showed that retroperitoneal laparoscopic partial adrenalectomy is safe when strict selection criteria and perioperative management are followed. The procedure potentially shortens the hospital stay and reduces hospitalization costs.

Patients with PCa develop resistance to vinblastine, and Chen et al. aims to explore the genetic alterations underlying this resistance. The study identified CCNB1 and AURKA as critical genes for PCa progression and castration-resistant PCa resistant to vinblastine. The findings provide insights into the mechanisms of resistance and potential therapeutic targets for patients with PCa.

Zhou et al. explores the relationship between C-reactive protein (CRP) levels and PCa prognosis. Elevated CRP levels were associated with worse overall survival, cancer-specific survival, and progression-free survival in prostate cancer patients. The study suggests that CRP levels could serve as a prognostic marker for PCa outcomes, though further validation is needed.

Li et al. compares the efficacy and safety of conservative treatment and surgery for patients with small nonfunctional adrenal incidentalomas. The results indicate that surgical treatment is more effective for 1-3cm nonfunctional adrenal incidentaloma, but conservative treatment is safer and more economical. Follow-up after both treatments is essential.

Li et al. compares tumor control in patients with PCa with oligometastasis following combined robot-assisted radical prostatectomy and androgen deprivation with androgen deprivation therapy alone using PSA percentage decline rate. The study concludes that both treatments provide effective tumor control, with combined therapy showing a significant advantage in PSA percentage decline rate without compromising urinary continence.

Li et al. identifies hub genes related to bicalutamide resistance in prostate cancer and constructs a prognostic model for patient prognosis. Genetic alterations associated with resistance to bicalutamide are explored, providing potential targets for endocrine therapy resistance in prostate cancer patients.

You et al. investigates the efficacy and safety of bipolar androgen therapy in patients with castration-resistant PCa following resistance to abiraterone or enzalutamide. The review finds that bipolar androgen therapy is safe and effective, resensitizing patients to subsequent endocrine therapy and potentially improving overall survival and quality of life.

Wang et al. discusses surgical management strategies for complicated pheochromocytomas/paragangliomas based on case reports. The goal is to provide insights into the safe and effective surgical management of these tumors.

Yan et al. investigates whether intravenous volume expansion is necessary after preoperative α-blocker administration for adrenal pheochromocytoma. Analysis indicates that abandonment of intravenous volume expansion is not an independent risk factor for intraoperative hemodynamic instability. Tumor size is identified as an independent risk factor.

Liao et al. reports a rare case of adult bilateral pure androgen-secreting adrenal tumors (PASATs) and conducts a systematic review on adult PASATs. Characteristics of PASATs are summarized, including patient demographics, symptoms, androgen levels, tumor size, and malignancy features.

Li et al. compares the advantages and disadvantages of robot-assisted laparoscopic adrenalectomy with retroperitoneal laparoscopic adrenalectomy. robot-assisted laparoscopic adrenalectomy is found to reduce intraoperative blood loss and accelerate postoperative gastrointestinal recovery but has longer operation time, higher hospitalization expense, and different pathological types compared to retroperitoneal laparoscopic adrenalectomy.

Ren et al. aimed to develop a metabolic syndrome-related prognostic index (MSRPI) to predict biochemical recurrence-free survival (BFS) in patients with PCa and identify cold and hot tumors for personalized treatment. The researchers analyzed data from the Cancer Genome Atlas and Gene Expression Omnibus databases to identify prognostic metabolic syndrome-related genes and potential tumor subtypes. Using the LASSO algorithm and multivariate Cox regression, they constructed the MSRPI based on seven metabolic syndrome-related genes. The high-risk MSRPI was associated with poor BFS, validated internally and externally. The MSRPI also helped differentiate between cold and hot tumors, providing potential for precision therapy in patients with PCa.

Enzalutamide, a second-generation endocrine therapy drug for PCa, was analyzed to identify an enzalutamide-induced signature (ENZ-sig) for predicting progression and relapse-free survival in PCa by Feng et al. Candidate markers were derived from single-cell RNA sequencing analysis, and a 10-gene prognostic model (ENZ-sig) was constructed based on these markers. ENZ-sig was validated in multiple datasets, demonstrating its robust predictability. Biological enrichment analysis revealed that high ENZ-sig patients were more sensitive to cell cycle-targeted drugs, suggesting potential combination therapy strategies for PCa.

Gao et al. aimed to differentiate between adrenal Cushing syndrome (adrenal CS) and Cushing disease (CD) using serum steroid profiles. Liquid chromatography with tandem mass spectrometry was used to analyze 11 serum steroids in patients with adrenal CS or CD. The results showed distinct serum steroid profiles between adrenal CS and CD, with dehydroepiandrosterone sulfate, dehydroepiandrosterone, and androstenedione ratios identified as biomarkers for discrimination. The study highlights the diagnostic value of serum steroid profiles in distinguishing between adrenal CS and CD.

Du et al. aimed to explore how sex hormones affect the association between 25-hydroxyvitamin D [25(OH)D] and osteoporosis using a two-step Mendelian randomization (MR) analysis. The results showed that 25(OH)D and total testosterone (TT) had a causal effect on osteoporosis, while other sex hormones (A4, E2, and T/E2) did not show significant associations. The study provides evidence that TT mediates the causal effect of 25(OH)D on osteoporosis, suggesting that interventions targeting TT could reduce the burden of osteoporosis related to high 25(OH)D.

Huang et al. used MR analysis to explore the causal genetic association between thyroid function and benign prostatic diseases (BPDs). Genetic variations in thyroid function and BPDs were analyzed, and the results showed that TSH, subclinical hypothyroidism, and overt hypothyroidism had a significant effect on genetic susceptibility to benign prostate hyperplasia and prostatitis. However, hyperthyroidism and FT4 levels did not show significant effects. The study sheds light on the potential causal relationship between thyroid function and BPDs.

Liu et al. discusses the potential role of aldosterone, a mineralocorticoid hormone, in the pathogenesis of diabetic retinopathy (DR). Aldosterone may influence DR through its effects on oxidative stress, vascular regulation, and inflammatory mechanisms. The article highlights the value of aldosterone as a potential diagnostic and treatment target for DR, though further research is needed to fully understand the association between mineralocorticoids and DR.

Zhang et al. discusses the use of PARP inhibitors in PCa treatment. PARP inhibitors have shown promising results in patients with BRCA mutations. The article explores the potential of extending the use of PARP inhibitors to other populations, such as those with “BRCAness” tumors. Additionally, the interaction between androgen receptor signaling and PARP inhibitors is explored, providing insights into potential combination therapies for PCa.

Qin et al. investigated the impact of androgen deprivation therapy (ADT) on the tumor immune microenvironment in PCa. The analysis of single-cell transcriptomes from PCa tumors before and after ADT revealed a weakened killing effect of immune cells on tumors and reduced interaction between immune cells and tumor cells. The study also observed an increase in immunosuppressive cells (MDSC and Treg) after ADT. These findings suggest that ADT induces an immunosuppressive environment in the PCa tumor microenvironment, providing implications for combining ADT with immunotherapy.

Gao et al. aimed to establish and validate the cut-off values of estimated glomerular filtration rate (eGFR) and urinary albumin-to-creatinine ratio (UACR) for diabetic kidney disease (DKD). The study involved 374 patients with type 2 diabetes with baseline eGFR ≥60 mL/min/1.73 m2 and UACR <30 mg/g. The results showed that eGFR ≤84.8 mL/min/1.73 m2 or UACR ≥15.5 mg/g in the normal range may be effective cut-off values for predicting DKD.

Zhou et al. proposed a standardized scoring system, the ADRENAL score, to quantify the functional and anatomical characteristics of adrenal tumors. The score was based on six parameters, including functional status, tumor size, relationship to adjacent organs, intratumoral enhancement on CT, nearness to major vessels, and body mass index. The ADRENAL score was found to be a valid predictor of surgical outcomes in adrenal surgery, providing a common reference for preoperative risk assessment and comparative analysis of different surgical techniques and approaches.

## Author contributions

YS: Conceptualization, Writing – original draft, Writing – review & editing. CD: Writing – original draft. XL: Conceptualization, Writing – original draft, Writing – review & editing.
